# Traditional knowledge about plant, animal, and mineral-based remedies to treat cattle, pigs, horses, and other domestic animals in the Mediterranean island of Sardinia

**DOI:** 10.1186/s13002-018-0250-7

**Published:** 2018-07-20

**Authors:** Simonetta Bullitta, Giovanni Antonio Re, Maria Domenica Iole Manunta, Giovanna Piluzza

**Affiliations:** 1 Istituto per il Sistema Produzione Animale in Ambiente Mediterraneo - CNR-ISPAAM, Traversa La Crucca 3, località Baldinca, 07100 Sassari, Italy; 2 Present address: Wellcome Trust Centre for Cell Biology, University of Edinburgh, Edinburgh, UK

**Keywords:** Mediterranean ethno-veterinary, Plant remedies, Traditional therapeutics, Zoo-therapy, Livestock, Poultry, Pets

## Abstract

**Background:**

Mediterranean farmers traditionally utilized plants, animals, and minerals sourced locally to treat their animals. Research is needed to understand at what extent such knowledge of domestic animal care still survives and to document such traditions for further developments.

**Methods:**

We carried out our field study to recover ancient ethno-veterinary practices by means of questionnaires and interviews to farmers in rural areas of the Mediterranean island of Sardinia (Italy). Quantitative indices were used to evaluate the distribution and diversity of the acquired information.

**Results:**

We report here 98 sources (42 plant taxa, 14 animal-based substances, 15 minerals, and 27 other materials of various origin) emerged from the survey for the care of 41 ailments of cattle, pigs, and horses. Ethno-veterinary treatments, detailed in their formulations and applications, were used against ecto- and endo-parasites, gastrointestinal diseases, heart diseases, viral and bacterial diseases, wounds, sprains, and bruises.

**Conclusion:**

Our survey can be useful to implement the use of phyto-therapeutics and other remedies of non-herbal origin for diseased animals, and, as elderly farmers held most of the knowledge, it can contribute to the conservation of Mediterranean ethno-veterinary knowledge.

## Background

The knowledge and practices related to the use of medicinal plants for the treatment of human and animal diseases has been handed down from generation to generation in different cultures worldwide. In recent years, the traditional uses of numerous medicinal plants have been corroborated by scientific evidence [1]. The use of biological resources for medicinal purposes, however, is not restricted to human disease treatment, being also widely employed for treating diseases of livestock [2, 3]. These uses fall within the remit of ethno-veterinary medicine (EVM). The ethno-veterinary pharmacopoeia often contains ingredients sourced from various locations within the environment and may include plants, animals, and minerals [4].

In former times, the knowledge of medicinal plants was passed down orally from generation to generation; however, in modern Western societies of Europe, traditional knowledge is in danger of disappearing [5]. Ethno-veterinary surveys, on the preparation and utilization of herbal remedies have been conducted in Palestine, Latin America, Iran, Spain, Italy, Algeria, Morocco, Southern Italy, Brazil, Pakistan, India, and Polish-Lithuanian-Belarusian borderland [6–18].

The European Council Regulations on Organic Farming (nos. 834/2007 and 889/2008) [19] promote veterinary complementary medicine, i.e. phyto-therapeutic products, for the treatment of livestock diseases. Chemically synthesized allopathic veterinary medicines including antibiotics should only be used under the strict rules of Council Regulation (EC) no. 834/2007. There is an increasing demand for high-quality animal food products with no or limited use of pharmaceuticals produced either chemically or biotechnologically [6]. Ethno-veterinary data collected in the Mediterranean region can offer an extraordinary background for conducting studies aimed at implementing phytotherapy in animal health care and the use of plant-derived nutraceuticals, with the aim of improving the quality of animal-derived food products [20]. Many authors have argued that animals and/or their derivatives for medicinal use is a global phenomenon, dating back to prehistoric times and coevolving with human societies [21, 22]. In this respect, invertebrates and cognate products have been used worldwide to cure and/or prevent different human diseases [23–26]. The great interest around this group of animals, in particular insects, has grown due to their ability to synthesize a large number of chemical compounds [27]. Animals and products derived from their organs have constituted part of the inventory of medicinal substances used in different cultures since ancient times [24]. Despite its prevalence in traditional medical practices worldwide, research on medicinal animals, in comparison with medicinal plant research, has been often neglected [21, 28]; major emphasis have been put on medicinal plants because far more many species have been employed compared to medicinal animals. In addition, plants are somehow easier to collect, store, and trade. The importance of zoo-therapy in various socio-cultural environments around the world has been investigated [24]. A review on the ethno-veterinary use of invertebrates has revealed that humans have always considered this animal group as a source of surprising and extensive therapeutic properties [29].

Even though plants are at the core of ethno-veterinary medicine, other practices were also used, such as the use of drugs of animal origin and cauterization medicine.

The recovery of traditional plant knowledge (TPK) linked to their medicinal use is one of the most urgent and immediate issues needing attention, as confirmed by international researches. The preservation of popular traditions can contribute not only to identify new uses of plant species and to maintain ethno-biodiversity, but eventually to discover also novel biologically active compounds to treat diseases [30].

We have previously described ethno-veterinary treatments for small ruminants [13], here we point out the use of plants and their formulations for administration to cattle, horses, pigs, and dogs. In addition to plants, our study also revealed the use of further remedies of different nature and origin. Sardinian farmers utilized animals, minerals, and combinations of different materials to formulate remedies for their animals for prophylactic or therapeutic purposes.

### The study area

Cattle and pigs have been recorded in Sardinia since the Neolithic time; consequently, traditions of animal care date back to millennia. Cattle played an important role as working animals since the Nuragic period, during the Bronze Age, and this until the first part of the twentieth century, when draught animals were replaced by engines [31]. Horses were first introduced in Sardinia from Greece between the sixth and the fifth century B.C. [32]. The Roman Empire kept a breeding ground in the island for horses to be used in war and by gladiators; Saracen domination improved the Sardinian breeds crossing them with Arabian and Bedouin strains [33], and further breeding was developed towards the end of 1400 under the dominion of the Aragon Crown [33]. An intertwining of people, traditions, and knowledge about the care of domestic animals over the centuries makes the ethno-veterinary traditions of Sardinia peculiar and somehow unique. It is important to understand what is the current ethno-veterinary knowledge and at what extent plant, animal, and mineral substances are still in use in the traditional ethno-veterinary practices of Mediterranean areas.

Our aim was to perform a survey of Sardinian ethno-veterinary traditions not only those related to the use of plant species but also those involving other substances of animal or mineral origin and their combinations, in order to implement the studies on Mediterranean ethno-veterinary practices that are still poorly investigated. Our aim was also to understand which remedies were still in use and to document ethno-veterinary traditions to preserve them and prevent their unavoidable loss due to the oral way of transmission.

## Methods

### Ethnobotanical data collection

The investigation on traditional ethno-veterinary remedies was performed visiting Sardinian farmers and interviewing them individually at their farms. A questionnaire with open and closed questions was prepared according to Viegi et al. [34], with some modifications, as we aimed to recover all the ancient remedies of ethno-veterinary practices and not only those involving the use of plants. Our interviewees were asked to answer questions related to the type of illnesses and the animal species treated, to the preparation and the administration of the remedy, the frequency (current and past) of its use, and whether the same remedy was also employed for other purposes. The original forms filled for each remedy during the interviews are stored at CNR-ISPAAM.

**Fig. 1:**
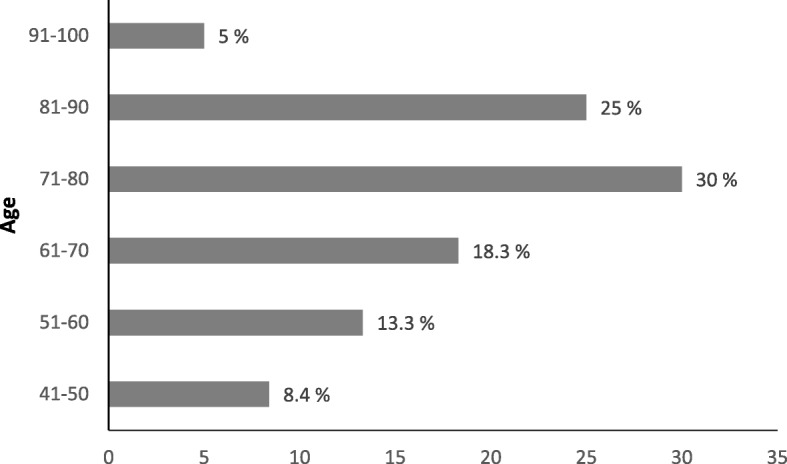
Percentage distribution of the interviewees into age groups

**Fig. 2:**
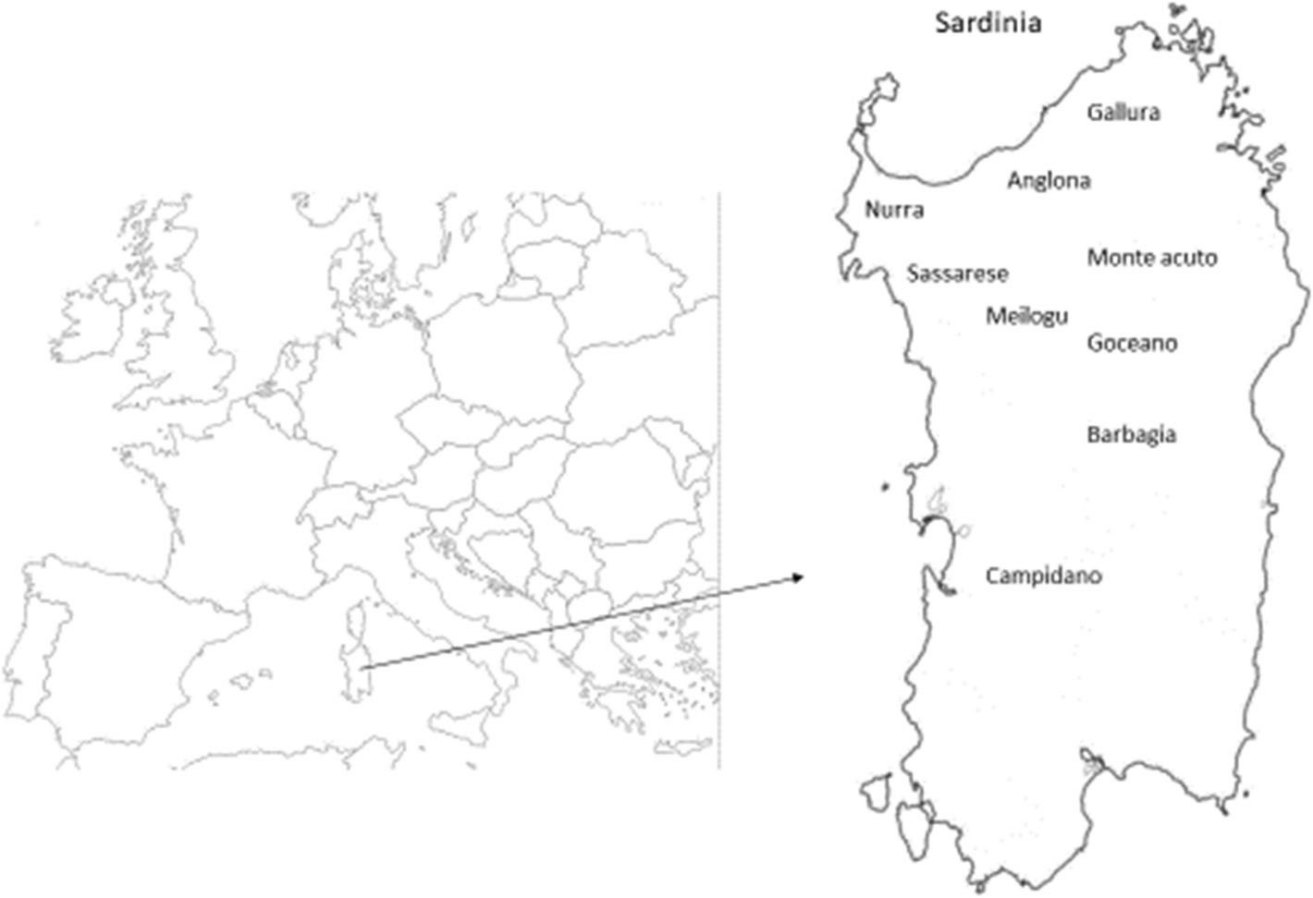
Map of Sardinia with the study areas

We interviewed 60 people, 50 men and 10 women, aged between 46 and 96 years old, being most of the participants between 61 and 80 years old (Fig. 1) with an average age ± standard deviation of 71.8 ± 13.7. All people were farmers and raised their animals in the Sardinian rural districts of Anglona, Barbagia, Campidano, Meilogu, Monte Acuto, Gallura, Goceano, Nurra, and Sassarese (Fig. 2). We paid particular attention on elderly people and to farms devoted to extensive animal breeding. We describe here remedies adopted for cattle, horses, pigs, poultry, dogs, and cats. According to the interviewees, most of the remedies were actively used between 1925 and 1985; however, considering that almost all stated to have learnt about the remedies from their parents or elderly relatives, it is likely that the remedies originated in earlier times.

Herbarium voucher specimens were collected during the interviews and are stored at CNR-ISPAAM in Sassari. The identity of plants was confirmed by classification according to Pignatti [35] and Conti et al. [33, 34, 36]; familial nomenclature follows the Angiosperm Phylogeny Group (APG IV) [37].

### Data analysis

Three indices were applied: the Cultural Importance index (CI), the Relative Frequency of Citation (RFC), and the Relative Importance Index (RI):

1. The Cultural Importance index (CI), takes into account the spread of use and the diversity of uses of each plant species, according to Tardio and Pardo-de-Santayana [38], and represents the sum of the proportion of interviewees that mention each species use,
  $$ {\mathrm{CI}}_s=\sum \limits_{u={u}_1}^{u_{NC}}\sum \limits_{i={i}_1}^{i_N}\raisebox{1ex}{${\mathrm{UR}}_{ui}$}\!\left/ \!\raisebox{-1ex}{$N$}\right. $$
  it represents the sum of all the use reports (UR) for the species divided by the number of interviewees (*N*).
2. The Relative Frequency of Citation (RFC) was calculated as follows: the number of interviewees indicating the use of the species, also defined as frequency of citation (FC), divided by the total number of the interviewees (*N*),
  $$ {\mathrm{RFC}}_s=\frac{{\mathrm{FC}}_s}{N}=\frac{\sum \limits_{i={i}_1}^{i_N}{\mathrm{UR}}_i}{N} $$
  where UR is the sum of the use report of the species regardless the category use of the species.
3. The Relative Importance Index (RI) according to Pardo-de-Santayana [39] takes into account the use categories.
  $$ {\mathrm{RI}}_s=\frac{{\mathrm{RFC}}_{s\left(\max \right)}+{\mathrm{RNU}}_{s\left(\max \right)}}{2} $$
  where RFC_*s*(max)_ is the relative frequency of citation over the maximum number of citation, obtained by dividing FCs by the maximum value in all the species of the survey. RNU_*s*(max)_ is the relative number of use categories over the maximum, obtained by dividing the number of uses of the species by the maximum value in all the species in the survey. The use categories were (a) ecto- and endo-parasite diseases, (b) gastrointestinal diseases and heart diseases, (c) viral and bacterial diseases, and (d) wounds, sprains, and bruises.

The survey was carried out taking into account the protection of biodiversity and the rights of local people according to the principles stated by [40, 41], in agreement with the principles of the International Society of Ethnobiology Code of Ethics (http://ethnobiology.net/code-of-ethics/).

Some of the ethno-veterinary practices here reported do not comply with the Italian national legislation for domestic animal welfare (D.L.146/2001) or European community regulations concerning the protection of animals kept for farming purposes (Council directive 98/58/EC). They are just reported and not endorsed by authors and although dismissed are mentioned for the sake of completeness of the survey.

## Results

### Quantitative analysis

**Fig. 3:**
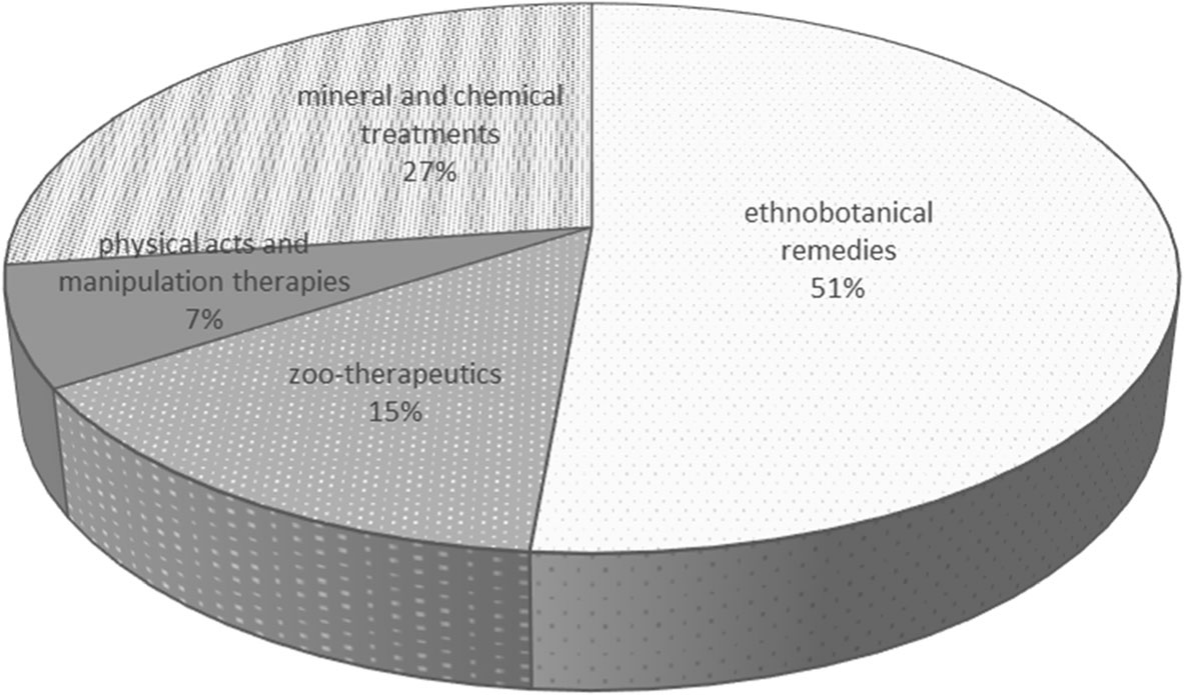
Percentage distribution of plant-, animal-, and mineral-based remedies

**Fig. 4:**
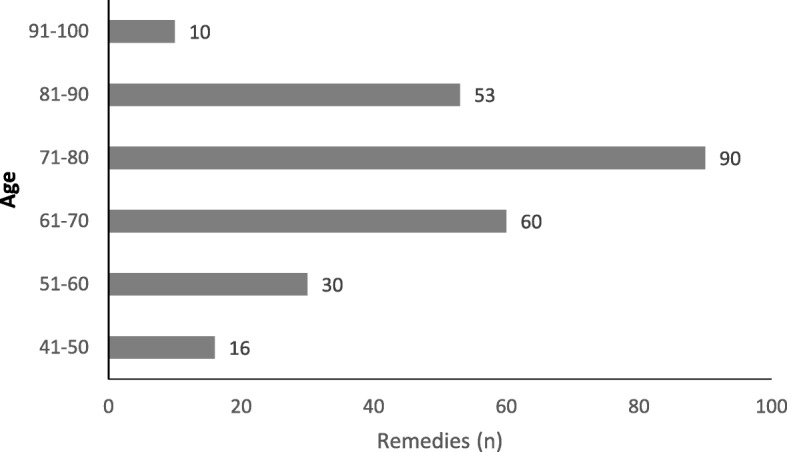
Number of recorded remedies according to the age group of the interviewees

We found that in the Sardinian traditional health care for domestic animals, the percentage of ethno-botanical remedies was 51.4% while zoo-therapeutics accounted for 14.4%, physical acts and manipulation therapies were 7%, and the mineral and chemical treatments were 27.2%. (Fig. 3). No magic rituals were mentioned by our interviewees. The highest number of remedies (90) was reported by the participants aged between 71 and 80 years (Fig. 4).

The identified traditional ethno-veterinary remedies were used to treat cattle, horses, pigs, dogs, cats, and hens against ecto- and endo-parasites, gastrointestinal diseases, heart diseases, viral and bacterial diseases, wounds, sprains, and bruises. Ninety-eight sources were documented in this survey, including: 42 plant taxa, 14 animal derivatives, 15 minerals, and 27 other materials of various origins. The herbal remedies included 30 spontaneous plant species, quite widespread in the Sardinian pasturelands, 11 cultivated species (onion, garlic, oat, parsley, tobacco, barley, wheat, broad beans, lineseed, olive, vine), and 1 ornamental (camellia). The plants mentioned belonged to 29 botanic families. The most represented were Poaceae with five species, Apiaceae with four species, followed by Leguminosae, Malvaceae, Urticaceae, Asteraceae, and Fagaceae with two species each. Plant-derived products such as olive oil, vinegar, beer, and cork were also used alone or in combination with other substances to prepare remedies. Plant species and their ethnobotanical indices are listed in Table 1. The ranking according to each index (Table 1) shows that the species *Olea europaea* L., *Vitis vinifera* L., *Malva sylvestris* L., *Hordeum vulgare* L., *Parietaria officinalis* L., *Pistacia lentiscus* L., *Matricaria chamomilla* L., and *Triticum durum* Desf. were in the first eight positions due to their higher indices. The species *Vitis vinifera* and *Olea europaea* which ranked in the first two positions for CI, RI, RFC were among the most cited (26 and 22 interviewees, respectively) for the treatments of 6 and 8 ailments. The local importance of each species calculated by using the Relative Frequency of Citation (RFC) showed that *Vitis vinifera* (RFC 0.43), *Olea europaea* (RFC 0.37), and *Malva sylvestris* (RFC 0.33) represent the core of the cultural ethnobotanical heritage in the investigated areas (Table 1). The same table shows the RI index of plant species. *Vitis vinifera* (RI 1) was employed in all the four use categories. *Olea europaea* (RI 0.80), *Malva sylvestris* (RI 0.76), *Parietaria officinalis* (RI 0.57), *Pistacia lentiscus* (RI 0.55), and *Triticum durum* (RI 0.51) were employed in three of the four use categories. They showed higher RI values compared to the other plant species with RI values ranging from 0.38 to 0.14 and employed for two or one use categories.

**Table 1:** Quantitative indices of plant species: CI (cultural importance); RI (relative importance); RFC (relative frequency of citation)

Voucher specimen	Species (Family)^a^	Local names	Indices			Ranking		
			CI	RI	RFC	CI	RI	RFC
GPE13	*Olea europaea* L. (Oleaceae)	Olìa	0.35	0.80	0.37	1	2	2
GPE36	*Vitis vinifera* L. (Vitaceae)	Bide	0.35	1	0.43	2	1	1
GPE11	*Malva sylvestris* L. subsp. *sylvestris* (Malvaceae)	Pramuzza	0.25	0.76	0.33	3	3	3
GPE30	*Hordeum vulgare* L. (Poaceae))	Ozu	0.12	0.38	0.12	4	7	6
GPE16	*Parietaria officinalis* L. (Urticaceae)	Pigulosa	0.10	0.57	0.17	5	4	4
GPE18	*Pistacia lentiscus* L. (Anacardiaceae)	Chessa	0.10	0.55	0.15	6	5	5
GPE12	*Matricaria chamomilla* L. (Asteraceae)	Caboniglia	0.08	0.35	0.08	7	8	9
GPE38	*Triticum durum* Desf. (Poaceae)	Trigu	0.08	0.51	0.12	8	6	7
GPE01	*Allium cepa* L. (Amaryllidaceae)	Chibudda	0.05	0.24	0.10	9	14	8
GPE33	*Quercus pubescens* Willd. (Fagaceae)	Chelcu	0.05	0.31	0.05	10	10	12
GPE23	*Umbilicus rupestris* (Salisb.) Dandy (Crassulaceae)	Calighe de muru	0.05	0.33	0.07	11	9	11
GPE02	*Allium sativum* L. (Amaryllidaceae)	Azu	0.03	0.29	0.03	12	11	14
GPE43	*Apium nodiflorum* Lag. (Apiaceae)	Apieddu	0.03	0.29	0.03	13	12	15
GPE25	*Calamintha nepeta* (L.) Savi (Lamiaceae)	Nebida	0.03	0.20	0.07	14	15	10
GPE39	*Linum usitatissimum* L. (Linaceae)	Linu	0.03	0.16	0.03	15	18	17
GPE34	*Quercus suber* L. (Fagaceae)	Suerzu	0.03	0.27	0.02	16	13	22
GPE24	*Urtica dioica* L. subsp. *dioica* (Urticaceae)	Pistija	0.03	0.18	0.05	17	16	13
GPE48	*Anagyris foetida* L. (Leguminosae)	Giolva	0.02	0.14	0.02	18	23	23
GPE04	*Arundo donax* L. (Poaceae)	Canna	0.02	0.14	0.02	19	24	24
GPE44	*Avena sativa* L. (Poaceae)	Aena	0.02	0.14	0.02	20	25	25
GPE49	*Camellia sp.* L. (Theaceae)	Camelia	0.02	0.14	0.02	21	26	26
GPE06	*Cistus creticus* L. subsp. *eriocephalus* (Viv.) Greuter et Burdet (Cistaceae)	Mudeju	0.02	0.14	0.02	22	27	27
GPE41	*Citrus limon* L. (Osbeck) (Rutaceae)	Limoni	0.02	0.14	0.02	23	28	28
GPE29	*Daphne gnidium* L. (Thymelaeaceae)	Patteddu	0.02	0.14	0.02	24	29	29
GPE37	*Daucus carota* L. (Apiaceae)	Pistinaca	0.02	0.14	0.02	25	30	30
GPE07	*Dipsacus fullonum* L. (Caprifoliaceae)	Cardu aresti	0.02	0.14	0.02	26	31	31
GPE08	*Euphorbia characias* L. (Euphorbiaceae)	Lattorigu	0.02	0.14	0.02	27	32	32
GPE09	*Ficus carica* L. var. *caprificus* (Moraceae)	Crabufigu	0.02	0.14	0.02	28	33	33
GPE40	*Lavatera olbia* L. Alef. (Malvaceae)	Prammutza ‘óina	0.02	0.16	0.03	29	17	16
GPE47	*Nasturtium officinale* R.Br. (Brassicaceae)	Ascione	0.02	0.14	0.02	30	34	34
GPE31	*Nicotiana tabacum* L. (Solanaceae)	Tabaccu	0.02	0.16	0.03	31	19	18
GPE15	*Opuntia ficus indica* L. (Cactaceae)	Figuindia	0.02	0.14	0.02	32	35	35
GPE17	*Petroselinum crispum* (Mill.) Nym. (Apiaceae)	Petrusimula	0.02	0.16	0.03	33	20	19
GPE51	*Plantago major* L. (Plantaginaceae)	Nerviadile	0.02	0.14	0.02	34	36	36
GPE19	*Prunus spinosa* L. subsp. *spinosa* (Rosaceae)	Pruniskedda	0.02	0.16	0.03	35	21	20
GPE20	*Pteridium aquilinum* (L.) Kuhn (Hypolepidaceae)	Filighe	0.02	0.16	0.03	36	22	21
GPE21	*Sambucus nigra* L. (Adoxaceae)	Sambuccu	0.02	0.14	0.02	37	37	37
GPE46	*Santolina chamaecyparissus* L. (Asteraceae)	Santulina	0.02	0.14	0.02	38	38	38
GPE22	*Smilax aspera* L. (Smilacaceae)	Tetti	0.02	0.14	0.02	39	39	39
GPE42	*Smyrnium olusatrum* L. (Apiaceae)	Lisandru	0.02	0.14	0.02	40	40	40
GPE35	*Vicia faba* L. (Leguminosae)	Fae	0.02	0.14	0.02	41	41	41
GPE45	*Zea mays* L. (Poaceae)	Triguìndia	0.02	0.14	0.02	42	42	42

^a^(Familial nomenclature follows the Angiosperm Phylogeny Group (APG IV)

The non-herbal remedies (Table 2) involved the use of substances such as lard, salt, ashes, *ozzu casu* (fat obtained by boiling the cream of milk with flour), copper sulphate, *ozzu brujadu* (reused motor oil), *ozzu porchinu* (fat from lard), and *ozzu seu* (dried peritoneum of sheep). According to the RFC index (Table 3), the most locally important among the sources different from plants were lard (RFC 0.35), salt (RFC 0.23), and ashes (RFC 0.22). As shown on Table 2, salt was cited by 14 interviewees for treating seven diseases in cattle and horses; the use of ashes in nine different remedies was indicated by 13 interviewees for the treatment of six diseases occurring in cattle, horses, and pigs. Among the animal-derived substances, the lard usage was cited by 21 interviewees as component of 11 different remedies to treat four diseases affecting cattle and horses. The highest values for the CI index of sources different from plants (Table 3) were found for lard (CI 0.32), salt (CI 0.20), copper sulphate (CI 0.19), *ozzu casu* (CI 0.19), and ashes (CI 0.16). According to the RI of such sources, lard (RI 0.60), salt (RI 0.53) and *ozzu casu* (RI 0.48), employed in three of the four use categories, showed higher values, compared to other 11 sources which showed RI ranging from 0.44 to 0.27 (clay, ashes, copper sulphate, *ozzu brujadu*, cuttlefish bone, *ozzu seu*, sugar, knife, *ozzu porchinu*, seawater, cow’s milk) and which were employed in only two of the four use categories. Lard and salt were used for ailments included in the use categories of gastrointestinal diseases, viral and bacterial diseases, and wounds, sprains, and bruises, while *ozzu casu* was reported for ailments in the use categories of ecto- and endo-parasite diseases, viral and bacterial diseases, and wounds, sprains, and bruises.

**Table 2:** Non-herbal sources of remedies and their uses

Sources	Remedies (no.)	Ailments (no.)	Interviewees (no.)	Animal treated
Lard	11	4	21	Cattle ^a^, horses
Salt	10	7	14	Cattle ^a^, horses
Ashes	9	6	13	Cattle ^a^, horses, pigs
Copper sulphate	8	3	11	Cattle ^a^, horses, pigs, dogs, cats, hens
Clay	6	5	6	Cattle ^a^, horses, little pigs
*Ozzu casu* (fat obtained by boiling the milk cream with flour)	6	5	10	Cattle ^a^, horses, pigs, dogs, cats, hens
*Ozzu seu* (dried peritoneum of sheep)	5	3	6	Cattle ^a^, dogs
Brewer’s yeast	4	4	10	Cattle ^a^, horses
Cuttlefish bone (*Sepia officinalis* L.)	3	2	6	Cattle ^a^, horses, pigs
*Frammentalzu* (mother yeast for bakery)	3	1	3	Cattle ^a^
*Ozzu porchinu* (fat from lard)	3	2	3	Pigs, cows
*Ozzu brujadu* (reused motor oil)	3	2	7	Pigs, oxen
Knife	3	3	3	Cattle ^a^
Scissors	3	1	4	Cattle ^a^
Sugar	3	2	4	Cattle ^a^
Urine	3	2	3	Cows, pigs
Beeswax	2	1	2	Cows
Brine	2	1	2	Cattle ^a^
Creolin	2	1	2	Horses, pigs
Cow’s milk	2	2	2	Cattle ^a^, pigs
Naphtha (diesel oil)	2	2	3	Cattle ^a^
Needle	2	1	6	Cows, oxen
Seawater	2	2	3	Cattle ^a^, horses
Soap	2	2	2	Cows, oxen
Warm water	2	2	2	Cattle ^a^, cat
A bath in the river	1	1	1	Oxen
Acetylsalicylic acid	1	1	2	Horses
Beer	1	1	4	Cattle ^a^
Blood of rabbit	1	1	1	Pigs
Butter	1	1	1	Cattle ^a^
Cicatrene	1	1	1	Horses
Coal	1	1	1	Horses
Coffee	1	1	2	Cattle ^a^
Coke	1	1	1	Cattle
Ethyl alcohol	1	1	1	Pigs
Iodine	1	1	1	Cows
Lead acetate	1	1	1	Horses
Leech (*Hirudo medicinalis* L.)	1	1	1	Cattle ^a^
Lime	1	1	1	Cattle ^a^
Goat milk	1	1	1	Pigs
Mud	1	1	1	Cattle ^a^, horses, pigs, dog, cats, hens
Peg	1	1	1	Cows
Penicillin	1	1	1	Cows
Petroleum	1	1	2	Cattle^a^
Pig tail	1	1	1	Pigs
Pins	1	1	1	Oxen
Pumice stone	1	1	2	Pigs, dogs
Red hot iron	1	1	3	Cattle^a^, horses
Galloping	1	1	1	Horses
Red hot spike	1	1	1	Horses
Rope made of goat’s hair	1	1	1	Horses
Rough stone	1	1	1	Pigs
Silver coin (Five liras)	1	1	1	Cows
Warm clothes	1	1	1	Horses
Waxed thread	1	1	1	Pigs
Wet clothes	1	1	1	Cattle^a^

^a^Cure for cows, calves, and oxen

**Table 3:** Quantitative indices of sources other than herbal: CI (cultural importance); RI (relative importance); RFC (relative frequency of citation)

Sources	Indices			Ranking		
	CI	RI	RFC	CI	RI	RFC
Lard	0.32	0.60	0.35	1	1	1
Salt	0.20	0.53	0.23	2	2	2
Copper sulphate	0.19	0.37	0.18	3	6	4
*Ozzu casu* ^a^	0.19	0.48	0.17	4	3	6
Ashes	0.16	0.39	0.22	5	5	3
Brewer’s yeast	0.14	0.23	0.17	6	15	5
*Ozzu brujadu* ^b^	0.10	0.33	0.12	7	7	7
Clay	0.09	0.44	0.10	8	4	8
Cuttlefish bone	0.09	0.32	0.10	9	8	9
Needle	0.09	0.19	0.10	10	16	10
*Ozzu seu* ^c^	0.09	0.32	0.10	11	9	11
Scissors	0.06	0.17	0.07	12	18	13
Seawater	0.06	0.28	0.05	13	13	19
Sugar	0.06	0.29	0.07	14	10	14
*Frammentalzu* ^d^	0.04	0.16	0.05	15	19	15
Knife	0.04	0.28	0.05	16	11	16
*Ozzu porchinu* ^e^	0.04	0.28	0.05	17	12	17
Red hot iron	0.04	0.16	0.05	18	20	18
Urine	0.04	0.16	0.05	19	21	20
Acetylsalicylic acid	0.03	0.15	0.03	20	22	21
Beeswax	0.03	0.15	0.03	21	23	22
Brine	0.03	0.15	0.03	22	24	23
Coffee	0.03	0.15	0.03	23	25	24
Creolin	0.03	0.15	0.03	24	26	25
Cow’s milk	0.03	0.27	0.03	25	14	26
Petroleum	0.03	0.15	0.03	26	27	27
Soap	0.03	0.15	0.03	27	28	29
Water	0.03	0.15	0.03	28	30	30
A bath in the river	0.01	0.14	0.02	29	31	31
Beer	0.01	0.17	0.07	30	17	12
Blood of rabbit	0.01	0.14	0.02	31	32	32
Butter	0.01	0.14	0.02	32	33	33
Cicatrene	0.01	0.14	0.02	33	34	34
Coal	0.01	0.14	0.02	34	35	35
Coke	0.01	0.14	0.02	35	36	36
Ethyl alcohol	0.01	0.14	0.02	36	37	37
Galloping	0.01	0.14	0.02	37	38	38
Iodine	0.01	0.14	0.02	38	39	39
Lead acetate	0.01	0.14	0.02	39	40	40
Leech (*Hirudo medicinalis*)	0.01	0.14	0.02	40	41	41
Lime	0.01	0.14	0.02	41	42	42
Goat milk	0.01	0.14	0.02	42	43	43
Mud	0.01	0.14	0.02	43	44	44
Naphtha (diesel oil)	0.01	0.14	0.02	44	45	45
Peg	0.01	0.14	0.02	45	46	46
Penicillin	0.01	0.14	0.02	46	47	47
Pig tail	0.01	0.14	0.02	47	48	48
Pins	0.01	0.14	0.02	48	49	49
Pumice stone	0.01	0.15	0.03	49	29	28
Red-hot spike	0.01	0.14	0.02	50	50	50
Rope made of hair (from goat)	0.01	0.14	0.02	51	51	51
Rough stone	0.01	0.14	0.02	52	52	52
Silver coin (Five liras)	0.01	0.14	0.02	53	53	53
Warm clothes	0.01	0.14	0.02	54	54	54
Waxed thread	0.01	0.14	0.02	55	55	55
Wet clothes	0.01	0.14	0.02	56	56	56

^a^Fat obtained by boiling the milk cream with flour

^b^Reused motor oil

^c^Dried peritoneum of sheep

^d^Mother yeast for bakery

^e^Fat from lard

**Fig. 5:**
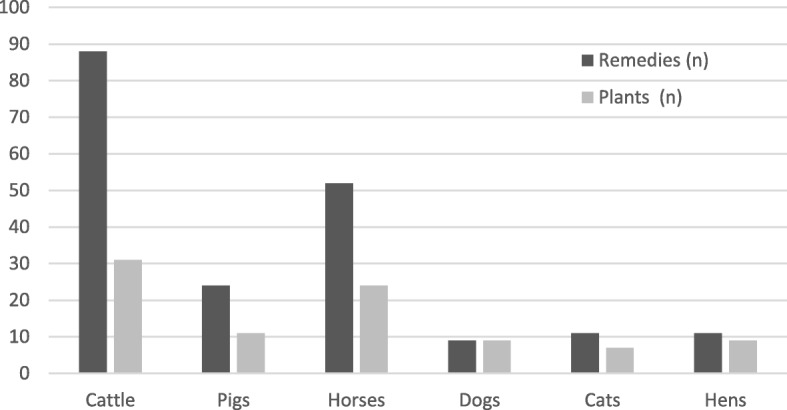
Number of plant species and related remedies used for the care of each animal species

**Fig. 6:**
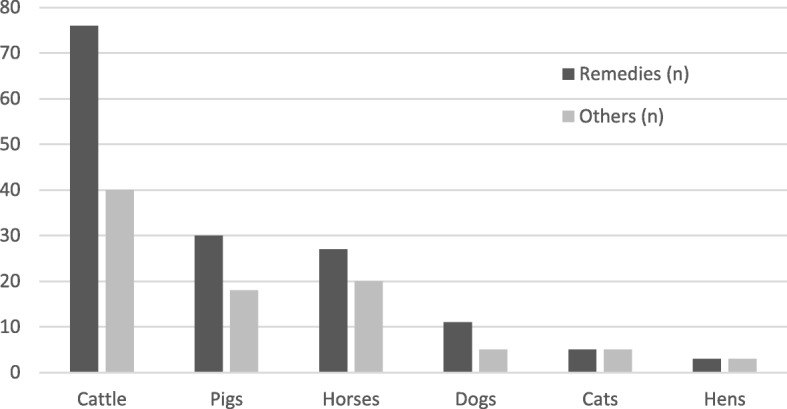
Number of substances of non-herbal origin (Others) and related remedies used for the care of each animal species

**Fig. 7:**
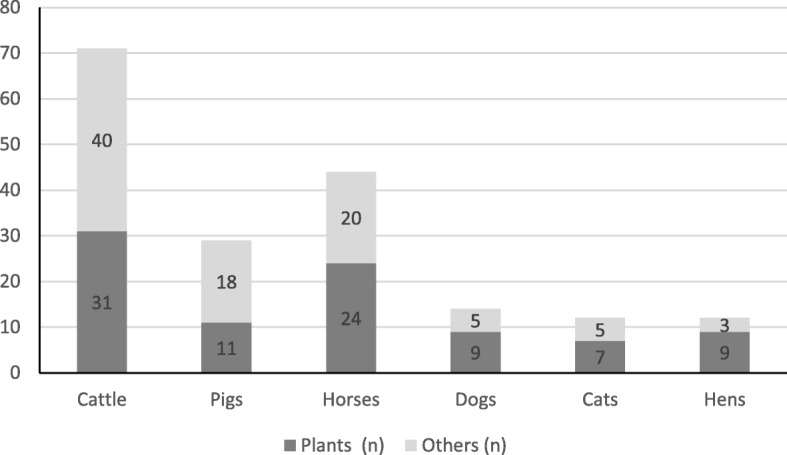
Comparison of the number of plant species (Plants) and substances of non-herbal origin (Others) used for the care of each animal species

The highest number of plant species and related remedies were used in the care of cattle (Fig. 5) as well as the highest number of non-herbal components and related remedies of non-herbal origin (Fig. 6). As shown in Fig. 7, horses, dogs, cats, and hens were prevalently treated with remedies of botanical origin while remedies from other sources outnumbered those of botanical origin for the treatment of pigs and cattle.

### Ethno-veterinary treatments

The ethno-veterinary procedures against ecto- and endo-parasites are listed in Table 4, the ones still in use are marked with an asterisk. Burnt cork, olive, and lentisk oil were scrubbed on skin in the treatment of mange. Non-herbal remedies were also described, involving *ozzu porchinu*, copper sulphate, *ozzu casu*, *ozzu seu*, *ozzu brujadu*, pomice stone, and diesel oil. Olive oil was also used in the treatment of lice and forest flies in cattle and horses (Table 4). The remedies for the treatment of foot rot were only non-herbal: seawater, lime, *ozzu seu*, and copper sulphate. Eight out of the 28 remedies against ecto- and endo-parasites indicated in Table 4 are still in use, mainly on pigs or dogs, only two are based on plants, plum leaves to treat wounds infected by maggots in cattle and horses, and burnt cork for mange in dogs.

**Table 4:** Ethno-veterinary remedies against ecto- and endo-parasites

Animals	Components of remedy^**^	Procedure	Areas
Mange			
Pigs, cows	**Lentisk oil**	Scrubbed on skin	Gallura
Pigs	(*) *Ozzu porchinu* (fat from lard)	Mixed, scrubbed on nose	Monte Acuto
Dogs	**Copper sulphate**	Scrubbed on infested skin	Goceano
Dogs	**Copper sulphate**, **olive oil**	Scrubbed on infested skin	Nurra
Pigs, dogs	(*) **Copper sulphate**, ***ozzu casu*** (fat obtained by boiling the cream of milk with flour), pumice stone	The skin was scrubbed using a pumice stone prior applying the mixture	Sassarese, Nurra
Dogs	(*) **Copper sulphate**, ***ozzu seu*** (dried peritoneum of sheep)	Mixed, scrubbed on nose	Goceano
Dogs	(*) Burnt cork	Scrubbed on nose	Goceano
Cows	Albanian spurge (*Euphorbia characias*) stems	Stems of the plant applied on the infected skin	Sassarese
Pigs	(*) **Olive oil**	Scrubbed on skin	Sassarese
Pigs, oxen	(*) ***Ozzu brujadu*** (Reused motor oil)	Applied on the skin with a brush	Monte Acuto, Sassarese, Nurra, Gallura, Anglona
Pigs	Seed oil, **copper sulphate**	Scrubbed on skin	Nurra
Pigs	**Cuttlefish** (*Sepia officinalis*) bone	The powder scrubbed on skin	Sassarese
Pigs	Diesel oil	Applied on the skin	Nurra
Cattle ^a^, horses, pigs, dogs, cats, hens	Dregs of **olive oil**, **copper sulphate**	Applied on the skin	Campidano di Oristano
Lice			
Cattle ^a^	**Olive oil**	Applied on the skin	Gallura
Hens	Lesser calmint (*Calamintha nepeta*)	The plant was placed in the hen house so that the smell kept away lice	Sassarese
Forest fly (*musca caddina*)(*Hippobosca equina* L.)			
Horses, cows	**Vinegar, olive oil**	Applied to the skin	Monte Acuto
Cattle ^a^	(*) **Olive oil**	Applied to the skin	Gallura
Cattle, horses, pigs, dogs, cats, hens	Navelwort (*Umbilicus rupestris*) leaves	Crushed fresh leaves applied onto the wound	Gallura
*Su solde* (Wounds infected by maggots)			
Cattle^a^, horses	(*) Plum tree (*Prunus spinosa*) leaves	Crushed fresh leaves applied onto the wound and wrapped with a bandage	Monte Acuto,
Cattle ^a^, pigs	Lesser calmint (*Calamintha nepeta*)	The fresh plants were smashed into a glass then the juice applied onto the wound	Monte Acuto, Meilogu
Cattle ^a^, horses, pigs	***Ozzu casu*** (fat obtained by boiling the cream of the milk with flour)	Massaged on wound	Monte Acuto
Foot rot			
Oxen	Knife	Needed to extract the worms	Monte Acuto
Horses	Seawater	Hoof washed with sea water	Gallura
Cattle^a^	Lime and water	Animals run through a foot bath	Gallura
Cattle ^a^	Hot ***ozzu seu*** (dried peritoneum of sheep)	Applied to the skin	Monte Acuto
cattle ^a^, pigs	**Copper sulphate**	Copper sulphate was ground and the powder was then applied to the foot	Sassarese, Anglona
Liver flukes			
Cattle ^a^	Brandy (distilled from **grapes**)	Given as a drink, administered as a preventive	Monte Acuto

^a^Cure for cows, calves, and oxen

(*)Remedies still in use

(**)Typed in bold are components of remedies showing highest indices in the quantitative analysis

As shown in Table 5, gastrointestinal diseases and heart diseases were predominantly treated with plant or plant-derived medicines and decoctions of plants given as feed (mallow, barley, wild carrot) or drink (olive oil, tree mallow). Gastrointestinal diseases were also treated in horses by applying warm clothes on the belly. Bloat was generally treated by non-herbal remedies such as lard, warm water, wet clothes, diesel oil, beer, and *frammentalzu* (mother yeast for bakery). Bloats in cows were treated also with ground lard given as feed, sometimes with the addition of parsley and onion, then a wet cloth was put on the animal. Brewer’s yeast dissolved in water was used in case of poisoning and as a refreshment in cattle. The ten remedies marked with an asterisk out of the 70 remedies for the treatment of gastrointestinal or heart diseases in Table 5 are still in use, mainly for cattle, and do not involve the use of plants except for onion mixed with lard for bloat treatment in cattle and hay for colics in cattle. Remedies for viral and bacterial diseases (Table 6) are predominantly of non-herbal origin; in the foot and mouth disease for example, pins were used to punch blisters in oxen, the seawater was used for mouth wash in cattle, and oxen were also soaked in the river for several days. Goat’s milk was administered intravenously to treat swine fever. The ashes were boiled in water and applied with a bandage, or dispersed in vinegar and used for manual udder massage in cattle mastitis. The powder of cuttlefish bone (*Sepia officinalis* L.) put into the eye or massaged around it, was a remedy to alleviate the pain of eye infection in cattle and horses. The burnt lard and burnt sugar were used to treat hoof infections in horses and oxen. A collar made of *Anagyris foetida* L. was placed around the neck of dogs with respiratory diseases, although these affections were also cured by using the decoction of either mallow or pellitory of the wall, or the fumes generated by burning leaves of wild fig trees. Only one of the 40 remedies against viral or bacterial diseases in Table 6 is still in use and it does not imply the use of plants. The ethno-veterinary remedies for treating wounds, gonadectomy, sprains, bruises, pimples, and swelling involved both the use of plants or substances of non-herbal origin (Table 7). Burnt lard, coal, acetylsalicylic acid, cicatrene, and *ozzu casu* were applied and massaged on the wounds in horses, pigs, bovines, and dogs. Ground fresh leaves of navelwort, elderberry, powder from stem, or the bark of lentisk was used to promote wound healing. To ease the effect of castration in pigs, the interviewees referred about the use of *ozzu brujadu* (reused motor oil), *ozzu casu*, urine, and ashes alone or added with olive oil; however, only one plant (mallow) was utilized to disinfect, heal, and soften the skin. Sprains and bruises were mainly cured with parts of plant or plant derivatives with the exception of sprains in cattle and equines where the cortex of *Quercus pubescens* Willd. was boiled with salt and vinegar, ground, mixed with clay, and then applied to the sore area wrapped with a bandage (Table 7). Skin lesions were treated with beeswax with or without the addition of *ozzu porchinu* and *ozzu seu*. To the swelling limbs of horses was applied clay alone or mixed with vinegar and salt, and lead acetate alone or added with water. Eight out of the 59 remedies indicated in Table 7 for the treatment of wounds, sprains, and bruises are still in use, mainly for cattle, horses, or dogs, three of them involved the use of plants, camellia for wounds in horses, and sarsaparilla and greater plantain for pimples in calves and fissures in cows. Further, farmers’ traditional uses of plants are shown in Table 8, and among them, ivy leaves are given to cows after giving birth, and stems of *Euphorbia characias* L. are used for catching eels. Twenty-seven of the reported remedies were still in use, those marked with an asterisk in Tables 4, 5, 6 and 7, mainly those employed for the treatment of gastrointestinal diseases, ecto- and endo-parasites, wounds, sprains, and bruises. The vast majority of the remedies was for topical administration (61.2%); fewer (37.8%) were for internal use (e.g. swallowed), and only 1% of the treatments implied the exposure to fumes.

**Table 5:** Ethno-veterinary remedies against gastrointestinal diseases and hearth’s disease

Animal	Components of remedy^**^	Procedure	Areas
Gastrointestinal infection, colics, diarrhoea			
Cattle^a^	Wild carrot (*Daucus carota*) leaves	Decoction given as feed	Barbagia di Nuoro
Cattle ^a^, pigs	**Wheat** bran	Bran mixed with water and given as feed	Gallura, Sassarese
Cattle ^a^	**Wheat** bran, coal	Coal grinded and mixed with wheat bran	Gallura
Cattle ^a^	**Mallow** (*Malva sylvestris*), **chamomile** (*Matricaria chamomilla*), olive oil	Decoction given as feed	Anglona
Cats	Water, **salt**	Given to drink	Gallura
Cats, horses	**Olive oil**	Given to drink	Gallura
Cows, oxen	Linseed oil	Mixed with water and given to drink	Monte Acuto
Horses, cattle ^a^	(*) **Brewer’s yeast**, water	Yeast mixed with water, given to drink	Monte Acuto, Anglona
Horses	**Barley** (*Hordeum vulgare*), water	Barley flour boiled with water given as feed	Monte Acuto
Horses	Warm clothes	Warm clothes on belly	Monte Acuto
Little pigs (Piglets)	(*) Dry **clay**	Given as feed	Sassarese
Little pigs (Piglets)	Dry clay, **barley** (*Hordeum vulgare*) flour	Given as feed	Sassarese
Cattle ^a^	Blades of prickly pear (*Opuntia ficus-indica*)	Cut into pieces and given as feed	Sassarese
Cattle ^a^	**Vinegar**	Given to drink	Barbagia di Nuoro
Cattle^a^	Brandy (distilled from **grapes**)	Given to drink	Barbagia di Nuoro
Oxen	**Pellitory of the wall** (*Parietaria officinalis*), water	Decoction of the plants filtered and given to drink	Monte Acuto
Cattle ^a^	(*) Hay	Given as feed	Nurra
Cattle ^a^, horses, pigs	**Mallow** (*Malva sylvestris*), water	Decoction of the plants filtered and given to drink	Gallura
Cows, oxen	**Mallow** (*Malva sylvestris*), water	Decoction of the plants filtered and given to drink	Monte Acuto
Oxen	Tree mallow (*Lavatera olbia*)	Decoction of the plant filtered and given to drink	Monte Acuto
Cattle ^a^	Fababeans (*Vicia faba*), **barley** (*Hordeum vulgare*), water	Beans and barley flour boiled in water given as feed	Anglona
Horses, oxen, calves	**Chamomile** (*Matricaria chamomilla*), water	Decoction of the plants filtered and given to drink	Meilogu, Goceano, Monte Acuto
Horses	Lemon (*Citrus limon*) juice, water	Decoction of juice given to drink	Goceano
Cattle ^a^	Flax-leaved daphne (*Daphne gnidium*) berries	Some berries mixed with forage and given as feed	Gallura
*Abbentadura* (Bloat)			
Cattle ^a^	**Olive oil**	One liter of olive oil given to drink after 2 or 3 days of fasting	Gallura, Monte Acuto
Cattle ^a^	Rancid **olive oil**	Given to drink	Nurra
Cattle ^a^	Warm water	Given to drink	Monte Acuto
Cattle ^a^	Petroleum	Given to drink	Nurra
Cattle ^a^	**Mallow** (*Malva sylvestris*), **chamomile** (*Matricaria chamomilla*), **olive oil**	Decoction given as feed	Anglona
Cows	Milk, **salt**, **olive oil**	The mixture given to drink	Monte Acuto
Cattle ^a^	**Lard**, parsley (*Petroselinum crispum*), onion (*Allium cepa*)	Onion bulbs, parsley and lard chopped, mixed, and given as feed to promote burping	Monte Acuto
Cows, cattle ^a^	(*) **Brewer’s yeast**, water	Mixed and given to drink	Monte Acuto, Sassarese, Meilogu
Cows	**Lard**, **wine**, **vinegar**	Mixed and given as feed	Monte Acuto
Cattle ^a^	(*)**Lard**, onion (*Allium cepa*)	Mixed and given as feed to promote burping	Monte Acuto
Cattle ^a^	Onion (*Allium cepa*)	Crushed and given as feed	Monte acuto
Cattle ^a^	**Lard**	Crushed and given as feed	Gallura, Monte Acuto Sassarese, Meilogu, Anglona
Cows	**Lard**, wet clothes	The lard was crushed and given as feed then a wet cloth was put on the animal	Monte Acuto
Cattle ^a^	Naphtha (diesel oil)	Three quarters of a liter of naphtha given in a bottle	Monte Acuto
Cattle ^a^	(*) Rancid **lard**	Crushed and given as feed, to promote burping	Monte Acuto, Nurra
Cattle ^a^	(*) Beer	Given to drink, to promote burping	Nurra, Sassarese
Horses	**Mallow** (*Malva sylvestris*), **vinegar**, water	Decoction given to drink	Sassarese
Cattle ^a^	**Olive oil**, **boiled wine**	Mixed and given to drink	Monte Acuto, Gallura
Cattle ^a^	(*) *Frammentalzu* (mother yeast for bakery)	Dissolved in water and given to drink	Monte Acuto, Sassarese
Cattle ^a^	*Frammentalzu* (mother yeast for bakery), lard, olive oil	Crushed and given as feed	Monte Acuto
Cattle, horses, pigs, dogs, cats, hens	**Lentisk** (*Pistacia lentiscus*) wood	Used to swab after incision of the vein under the belly	Campidano di Oristano
Cows, cattle ^a^	**Pellitory of the wall** (*Parietaria officinalis*)	Decoction of plants filtered and given to drink	Sassarese
Horses	Galloping	Deflation occurred after the galloping of horses in a field	Goceano
Cows	**Olive oil**, milk, **salt**	Give to drink	Monte Acuto
Poisoning			
Cattle ^a^	(*) **Brewer’s yeast**, water	As feed supplement	Nurra
Oxen	**Mallow** (*Malva sylvestris*), water	Decoction of plants filtered and given to drink	Monte Acuto
Constipation			
Cattle ^a^, horses, pigs, dogs, cats, hens ^a^,	**Vinegar** and **olive oil**	Mixed and given to drink	Anglona
Cows	**Mallow** (*Malva sylvestris*), water	Decoction of plants filtered and given to drink	Barbagia di Orgosolo
Cattle ^a^	**Olive oil**	Given to drink	Anglona
Horses	**Lentisk** fruits and leaves, water	Decoction of fruits and some leaves given to drink	Monte Acuto
Refreshing			
Horses	*Smirnium olusatrum*	The plant was collected in the summer and administered as feed	Monte Acuto
Cattle ^a^	**Brewer’s yeast**, water	Mixed and given to drink	Nurra
Oxen	**Chamomile** (*Matricaria chamomilla*), water	Decoction of leaves given to drink	Monte Acuto
Oxen	**Pellitory of the wall** (*Parietaria officinalis*), water	Decoction of leaves given as beverage	Monte Acuto
Post-partum collapse			
Cattle ^a^	(*)**Wine**, sugar	Given to drink	Anglona
Cattle ^a^	Coffee, **wine**, sugar	Given to drink	Monte Acuto
Angina pectoris			
Horses	**Lard**, **olive oil**	Massaged on the chest	Meilogu
High blood pressure			
Cattle ^a^	Leech (*Hirudo medicinalis*)		Anglona
Lack of appetite			
Cattle ^a^	**Barley** flour with water or milk	Given to drink	Monte Acuto
Cows	Fool’s-water-cress (*Apium nodiflorum*)	Fresh plant given as feed	Monte Acuto
Indigestion			
Horses	**Barley** (*Hordeum vulgare*), avena (*Avena sativa*), corn (*Zea mays*), flax (*Linum usitatissimum*) seed, water, salt	Decoction of mixture given to drink	Sassarese
Cattle ^a^	**Olive oil**	One liter of olive oil after 2 or 3 days of fasting given to drink	Gallura
Intestinal worms			
Cattle ^a^	Garlic (*Allium sativum*), vinegar	Two cloves of crushed garlic in half a liter of vinegar given as feed	Anglona
Horses	Bracken (*Pteridium aquilinum*) root	Burnt fern root fumes were breathed by horses covered with a blanket	Monte Acuto
Horses	Lavender cotton (*Santolina chamaecyparissus*), **barley** (*Hordeum vulgare*),	The dried plants given as feed	Barbagia di Nuoro
Horses	Giant cane (*Arundo donax*) leaves	The leaves given as feed and after 4 days the horse was fine	Meilogu

^a^Cure for cows, calves, and oxen

(*) Remedies still in use

(**) Typed in bold are components of remedies showing highest indices in the quantitative analysis

**Table 6:** Ethno-veterinary remedies against viral and bacterial diseases

Animals	Components of remedy^**^	Procedure	Areas
Foot and mouth disease (aphtha)			
Oxen	Pins	Pinching the blister	Gallura
Oxen	Fool’s-water-cress (*Apium nodiflorum*)	Fresh plant massaged in the tongue	Anglona
Oxen	Watercress (*Nasturtium officinale*)	Fresh plant massaged in the tongue	Anglona
Cattle ^a^	Seawater	Mouth washes	Anglona
Cows, oxen	**Needle**	Blisters on tongue were stung with a needle	Monte Acuto
Cows	**Needle**, scissors, **salt**	The vein under the tongue was stung with a needle, then the blisters were cut with scissors and salt was added on the wounds	Monte Acuto, Gallura
Cows	(*) **Needle**, **salt**	Blisters of tongue was stung with a needle and added with salt	Monte Acuto
Cattle ^a^	**Vinegar**, **salt**	Blisters of tongue were cut with a knife (or with a scissors) and tongue was disinfected with the mixture	Monte Acuto
Oxen	**Vinegar**, **salt**	Mouth washes	Anglona
Oxen	River	Oxen bathed in the river for several times	Monte Acuto
Pigs, cows	**Barley** (*Hordeum vulgare*), water	Barley flour boiled with water given as feed	Monte Acuto
Oxen	**Vinegar**	Mouth washes	Meilogu
Cattle ^a^	Brine	Applied to the tongue	Gallura
Cattle ^a^	Brine, **vinegar**	Applied to the tongue with a cloth	Goceano
Cattle ^a^, horses, pigs, dogs, cats, hens	Mud	Applied to the tongue	Anglona
Swine fever			
Pigs	Milk goats	Intravenous injection	Monte Acuto
Pigs	Blood of rabbit	Intravenous injection	Barbagia di Nuoro
Fever			
Oxen	**Mallow** (*Malva sylvestris*)	Decoction of plants was filtered and given to drink	Monte acuto
Mastitis			
Cows	Peg	The mammary vein was excised and then left bleeding, the haemorrhage was stopped by pinching the vessel with a peg	Sassarese
Cattle ^a^	**Ashes**, water	Ashes boiled in water and applied with a bandage	Monte Acuto, Sassarese, Anglona
Cattle ^a^	**Vinegar**, ash	Massaged on udder	Monte Acuto
Cattle ^a^, horses, pigs, dogs, cats, hens	Downy cork (*Quercus pubescens*) cortex, water	Cortex boiled in water until reddish, then the water was used to wash the udder	Barbagia di Nuoro
Cattle ^a^	***Ozzu casu*** (fat obtained by boiling the cream of the milk with flour)	Massaged on udder	Sassarese, Nurra, Gallura
Eyes infection			
Cattle ^a^	**Cuttlefish** (*Sepia officinalis*) bone	Powder inserted into the eye or massaged around the eyes	Monte Acuto, Meilogu, Anglona
Cattle ^a^, horses	**Cuttlefish** (*Sepia officinalis*) bone	Powder inserted into the eye	Gallura, Sassarese
Cattle ^a^	Wild teasel (*Dipsacus fullonum*)	Eye washed with the plant decoction	Anglona
Hoof infection			
Horses, oxen	Garlic (*Allium sativum*)	Crushed garlic application after nail clipping	Sassarese
Horses, oxen	Burnt **lard**	Burnt lard application after nail clipping	Sassarese
Oxen	Burnt sugar	The sugar was burnt over the wound	Monte Acuto
Horses	Rope made of goat’s hair	Incision of the nail with a knife then hoof dressed with the hairy rope	Monte Acuto
Cattle ^a^	**Clay**, water	Applied on the hoof with a bandage	Gallura
Blood poisoning (septicemia)			
Cows	Knife	Bleeding by incising the neck vein	Goceano
Pigs	Cow’s milk	Intravenous injection	Monte Acuto
Carbuncle			
Horses, cattle ^a^	Red-hot iron	Cauterization of the vesicles	Barbagia di Nuoro, Goceano, Monte Acuto
Respiratory diseases			
Dogs	Anagyris (*Anagyris foetida*)	The plant was put as a collar to the cold affected dog	Barbagia di Nuoro
Calves, oxen	**Chamomile** (*Matricaria chamomilla*), water	Decoction of plants given as drink	Monte Acuto
Oxen	**Mallow** (*Malva sylvestris*)	Decoction of plants given as drink	Anglona, Meilogu,
Cattle ^a^	**Pellitory of the wall** (*Parietaria officinalis*), water	Decoction of leaves given as drink	Anglona
Oxen	Wild ficus tree (*Ficus carica* var., *caprificus*)	The oxen covered with a blanket had to breathe the fumes of burnt leaves of wild fig tree for 3 days	Monte Acuto
Horses	**Hot bran** (*Hordeum vulgare*, *Triticum durum*)	Decoction of plant given as feed	Gallura

^a^Cure for cows, calves, and oxen

(*) Remedies still in use

(**) Typed in bold are components of remedies showing highest indices in the quantitative analysis

**Table 7:** Ethno-veterinary remedies relative to wounds, sprains, and bruises

Animals	Components of remedy**	Procedure	Areas
Wounds			
Oxen	Field mushroom	Dry powder applied on the wound	Monte Acuto
Horses	Burnt **lard**	Massaged on the wound	Monte Acuto
Horses	Acetylsalicylic acid	Massaged on the wound	Monte Acuto, Sassarese
Horses	Coke	Massaged on the wound	Sassarese
Horses	Cicatrene	Bought at the pharmacy	Sassarese
Horses	(*) Camellia (*Camellia* sp.)	Decoction of the plant massaged on the wound	Barbagia di Nuoro
Horses	Downy oak (*Quercus pubescens*) cortex, water	Cortex boiled in water applied on the wound, which was then wrapped up with a bandage	Anglona
Oxen	Tobacco (*Nicotiana tabacum*) leaves	Massaged on the wound	Monte Acuto
Oxen	Navelwort (*Umbilicus rupestris*) leaves	Pounded fresh leaves applied to the wound	Monte Acuto
Cattle ^a^, horses, pigs, dogs, cats, hens	Elderberry (*Sambucus nigra*)	Pounded fresh leaves applied to the wound	Gallura, Anglona
Cattle ^a^, horses, pigs, dogs, cats, hens	Powder from stem (without bark) or bark from stem of **lentisk** (*Pistacia lentiscus*)	Stem powder or ground bark applied on the wound	Anglona, Monte Acuto
Cattle ^a^	Powder of bark from stem of **lentisk** (*Pistacia lentiscus*) salt	Applied on the wound	Sassarese
Cattle ^a^, horses, pigs, dogs, cats, hens	Navelwort (*Umbilicus rupestris*) leaves	Minced fresh leaves applied to the wound	Gallura
Horses, cattle ^a^	**Mallow** (*Malva sylvestris*), water, soap	The wound was washed with soap and water, then decoction of leaves or root applied on the wound, which was then wrapped with a bandage	Sassarese, Gallura
Cattle ^a^, horses, pigs, dogs, cats, hens	**Olive oil**	Applied on the wound	Anglona, Gallura
Pigs, cattle	***Ozzu casu*** (fat obtained by boiling the cream of milk with flour)	Applied on the wound	Campidano di Oristano
Pigs, dogs	(*) **Copper sulphate**, ***ozzu casu*** (fat obtained by boiling the cream of milk with flour), pumice stone	Applied on the wound	Nurra
Pigs	**Olive oil**, ashes	Mixture as emollient cream for wound treatments	Campidano di Oristano, Monte Acuto
Pigs, cows	**Lentisk oil**	Applied on the wound	Gallura
Cat	**Olive oil**	Applied on the wound	Gallura
Cattle ^a^	Butter	Applied on the wound	Monte Acuto
Castration			
Pigs	**Olive oil**	Applied to the skin with a paintbrush	Sassarese
Pigs	***Ozzu brujadu*** (Reused motor oil)	Applied to the skin with a paintbrush	Sassarese
Pigs	**Olive oil**, **ashes**	Mixture as emollient cream for wound treatments	Campidano di Oristano
Pigs	***Ozzu casu*** (fat obtained by boiling the cream of milk with flour)	Applied on the wound	Campidano di Oristano
Pigs	Urine, **ashes**	Applied on the wound	Monte Acuto
Pigs	Urine, piece of pig’s tail	Applied on the wound	Monte Acuto
Pigs	Ethyl alcohol (or creolin in water), cord, hot wax	Ethyl alcohol (or creolin in water) and then the wound was sutured with a waxed thread	Monte Acuto
Pigs, horses	**Ashes**	Applied on the wound	Anglona
Pigs	**Mallow** (*Malva sylvestris*),	Applied on the wound	Anglona
Horses	Creolin, water	Applied on the wound	Anglona
Sprains			
Cattle ^a^, horses	Downy oak (*Quercus pubescens*) cortex, **salt**, **vinegar**, **clay**	The cork boiled with salt and vinegar, crushed, then mixed with clay, applied to the sore area, wrapped with a bandage	Monte Acuto
Horses	**Pellitory of the wall** (*Parietaria officinalis*), **mallow** (*Malva sylvestris*), Nettle (*Urtica dioica*), water	Decoction and plants wrapped in a bandage on the sore part	Monte Acuto
Dogs	(*) Burnt cork	Applied to the wound	Goceano
Bruises			
Cattle ^a^, horses, pigs	Nettle (*Urtica dioica*)	Decoction and plants wrapped in a bandage on the sore part	Anglona
Cattle ^a^, horses, pigs	**Pellitory of the wall** (*Parietaria officinalis*)	Decoction and plants wrapped in a bandage on the sore part	Anglona
Cattle ^a^, horses, pigs	**Mallow** (*Malva sylvestris*), water	Decoction and plants wrapped in a bandage on the sore part	Anglona
Horses	**Mallow** (*Malva sylvestris*), water, **vinegar**	Decoction and plants wrapped in a bandage on the sore part	Sassarese
Wounds from saddle			
Horses	**Ashes**	Applied on the wound	Nurra, Gallura
Wound from yoke			
Oxen	Cistus (*Cistus monspeliensis*)	The leaves applied at the inner base of the horns	Monte Acuto
Oxen	Soap, water	The mixture applied at the inner base of the horns	Monte Acuto
Pimples (Furuncles)			
Cows	*Chijnada* (**ashes** and water)	Ashes boiled in water and then the filtrate applied on the pimple	Monte Acuto
Cows	Urine	As disinfectant	Monte Acuto
Cows	Soap, water	Soap boiled in water and then the filtrate applied on the pimple	Monte Acuto
Calves ^a^	(*) Sarsaparille (*Smilax aspera*)	Decoction of plants wrapped in a bandage and put on the pimples	Monte Acuto
Fissures			
Cows	(*) *Ozzu porchinu* (fat from lard), ***ozzu seu*** (dried peritoneum of sheep), beeswax	The mixture was boiled and stored in a jar until use	Monte Acuto
Cows	(*) Beeswax	Massaged around the nipple	Monte Acuto
Cows	(*) Greater plantain (*Plantago major*), ***ozzu seu*** (dried peritoneum of sheep)	The mixture was boiled and was used when milking	Monte Acuto
Cows	Tincture of iodine, *ozzu porchinu* (fat from lard), ***ozzu seu*** (dried peritoneum of sheep), penicillin	The mixture massaged on the udder	Monte Acuto
Swelling udder			
Cows	Silver coin (five liras)	Massaged on the udder	Monte Acuto
Swelling throat			
Pigs	Rough stone	The throat was rubbed	Monte Acuto
Hens	**Vinegar**, water	The mixture was applied on the throat	Anglona
Cattle ^a^	**Wheat bran**, water	Boiled brans placed in a bag and tied in the throat	Anglona
Swelling limbs			
Horses	**Clay**	Applied to the limbs	Sassarese
Horses	Lead acetate	Applied to the limbs	Sassarese
Horses	(*) **Clay**, **vinegar**, **salt**	The mixture applied to the limbs	Sassarese
Horses	**Clay**, **vinegar**, water	The mixture applied to the limbs	Monte Acuto
Horses	Lead acetate, water	The mixture applied to the limbs	Sassarese
Swelling shank			
Horses	Red hot pin	Puncture with an iron pin	Monte Acuto

^a^ Cure for cows, calves and oxen

(*) Remedies still in use

(**) Typed in bold are components of remedies showing highest indices in the quantitative analysis

**Table 8:** Other traditional uses of plants suggested by farmers

Materials	Uses	Areas
Tree wormwood (*Artemisia arborescens*) flowers	To prepare spirits	Anglona
Ivy (*Hedera helix*)	Given to cows after giving birth	Barbagia di Nuoro
Wheat (*Triticum durum*) bran	Given to pigs as feed	Gallura
Albanian spurge (*Euphorbia characias*) stems	Stems used for catching eels	Sassarese

## Discussion

We developed the discussion about Sardinian ethno-veterinary practices considering the sharing of knowledge with Mediterranean, European, and extra-European countries, the actual use of such practices, and the eventual validation in scientific literature of the components of remedies.

### Use of animal body parts and/or animal substances

The therapeutic properties and uses of marine invertebrates were well known in the ancient Greek world and early Byzantine times; in particular, pulverized cuttlefish bone has been used in various eye itches and diseases [42]. The same use of pulverized cuttlefish bone was referred in our study, and it is supported by a recent review on anti-inflammatory, immune-modulatory, and wound healing properties of mollusks [43].

A vast amount of literature about leech therapy exists; active substances in leeches to prevent blood coagulation and treat osteoarthritis and other ailments in humans have received considerable attention [44], and in our survey, leeches were used to treat cows having high blood pressure.

Pig fat (lard) is an important component of several remedies for skin conditions in southern Italy; in addition to its emollient properties, it is also reported to be a useful vulnerary agent in the treatment of both animals and humans [45]. In our survey, lard was used to treat mange on pigs, bloat on cattle, hoof infection, and wounds on horses. Similarly, it has been used in Brazil for scabies, skin diseases, welling, burns, and wounds [46, 47]. Sheep suet has been also used for many disorders, including inflammation, sprains, and swelling [47], while in our study *ozzu seu* (dried peritoneum of sheep) was indicated for mange, foot rot, and fissures. The same authors have reported the use of milk of goat to treat weakness and malnutrition; in Sardinia, it was used to treat swine fever. Goat milk cream mixed with the pounded roots of *Panicum turgidum* Forssk. was applied topically to treat deep wounds and fractures in Africa [48].

Urine has been reported [49] as wound disinfectant, and that from cows has been shown to possess antioxidant and antibacterial properties [49]; in our survey, its use was recommended for porcine gonadectomy and for bovine pimples. Beeswax has been suggested to be effective for skin, for digestive disorders, and snake bites [44]. In Spain [6, 29], beeswax was used for cracks in the udder of cows, similar to our interviewees that used it to treat fissures in cows.

The use of animal parts or animal-derived products (*ozzu seu*, lard, *ozzu porchinu*, *ozzu casu*) is still practiced in Sardinian ethno-veterinary preparations and seven out of the 27 remedies still in use included such components.

### Use of mineral substances

The use of copper sulphate has been reported in Southern Italy either as a ground powder or dissolved in vinegar or with water and salt applied to cracked hooves or to the chapped skin surrounding the hooves of livestock [45]. In our study, copper sulphate was used for the treatment of mange in dogs, cattle, and pigs, for foot rot in cattle and pigs, and for wounds in pigs and dogs. Kyrgyz (central Asia) people have used blue stone or copper sulphate, white clay, and solution of sodium chloride to disinfect either the oral cavity of animals affected by foot and mouth disease or their external wounds [50]. A solution of copper sulphate has been used as anti-septic for wounds, while combustible sulphur has been employed to treat scabies [50]. It has been attested the use of a solution of copper sulphate in water to kill intestinal parasites [51]. Clay added with salt has been indicated to treat mastitis in cattle in Romania [4]; in our study, that remedy was used to treat gastrointestinal diseases in weaner pigs, hoof infections in cattle, sprains in cattle and horses, and swelling limbs in horses.

We reported the use of mud in the treatment of foot and mouth disease, the same use has been made in India [51]. Studies have demonstrated that mud therapy lowers the levels of inflammatory mediators and has a positive effect on antioxidant condition; recent investigations on the action mechanism of these products explain the reason of the empirical use of mud since ages [52].

Remedies were used in Sahara region such as bitumen and exhaust engine oil (based on products made available with modernization and globalization) to treat mange, and as insecticides against tick and flea infestations; and also cauterizations performed with iron tools to treat mastitis, abscesses, and inflammations [53], likewise the remedies reported by Sardinian farmers in our study. Salt dissolved in warm water and its topical application to bruises, muscular pains, and rheumatisms has been reported in Albania [54]; in our survey, in addition to these usages, it was suggested also for gastrointestinal problems, for foot and mouth disease and for wounds.

Only six remedies containing mineral substances are still in use: copper sulphate for mange in dogs, and, together with pumice stone for wounds in pigs and dogs, clay for diarrheoa in piglets and swelling limbs in horses; *ozzu brujadu* for mange in pigs and oxen; salt for foot and mouth disease in cows. While other nine remedies still in use, instead of mineral substances, include natural components (cork, olive oil, brewer yeast, *frammentalzu*, hay, wine, sugar).

In the case of cauterization medicine, a hot iron was used for curative purposes [55]; this tradition still survives in the Mid-Eastern veterinary practice [7]. In our study, a red-hot iron was indicated for the treatment of carbuncle in horses and cattle.

### Use of plants or plant- derivatives

In our survey, we recorded 42 plant taxa and 116 ethno-veterinary preparations with plants or plant-derived products as ingredients. In the survey carried out in circum-Mediterranean areas (eight nations) within the RUBIA project, 136 ethno-veterinary preparations and 110 plant taxa used for traditional animal health care have been recovered [2]. Twenty-six of the plant taxa in our ethno-veterinary survey were not mentioned in the report of the RUBIA project. In the review of plants used in folk veterinary medicine in Italy, Viegi [56] does not mention 14 of the species we recorded in our ethno-veterinary survey. Among the Sardinian ethno-botanic traditions investigated by Atzei [57], the species *Apium nodiflorum* Lag., *Daucus carota* L., *Dipsacus fullonum* L., *Nasturtium officinale* R.Br., *Petroselinum crispum* (Mill.) Nym., *Prunus spinosa* L., and *Camellia* sp. were not mentioned for ethno-veterinary uses.

In Spain, the remedy for pneumonia in cattle consisted in burning the aerial part of *Lavandula pedunculata* (Mill.) Cav. with sugar, to generate smoke [58]; similarly, Sardinia respiratory diseases in oxen were treated by fumigations of leaves of *Ficus carica* L.var. *caprificus*. Topical application of *Euphorbia oxyphylla* Boiss. latex has been used to treat wounds in equines [58], while in our study Camellia, tobacco leaves, *Quercus pubescens* cortex, navelwort leaves, elderberry leaves, powder of bark from stem of lentisk, and mallow were used for the same purpose. We found that *Daphne gnidium* L. was a remedy for gastrointestinal diseases in cattle, while in Spain it has been used to treat lambs with diarrhoea [58].

Consistent to our finding, it has been reported that for the traditional matanza (slaughter of swine and preparation of hams and sausage) pig fattening was implemented by surgical castration [58]. Nowadays, gonadectomy is performed by qualified veterinarians, but in the past, it was a duty for the most experienced family members. However, the procedure is not devoid of complications, and to minimize the risk of infections and inflammation, the succulent leaves of *Umbilicus rupestris* (Salisb.) Dandy have been used in Spain [58]. *U. rupestris* is a plant widely used according to ethno-veterinary studies in the Mediterranean region [13, 14, 59]. Our survey showed that mallow or olive oil were used for the same purposes, alongside non-herbal treatments (urine, ash, *ozzu casu*, reused motor oil), whereas *U. rupestris* was employed for other types of wounds in cattle, horses, pigs, dogs, cats, and hens. The use of *Malva sylvestris* in the management of gastrointestinal diseases has been shown to be a quite broadly diffused practice in Spain [58] and Argentina [18], and our findings reported the same use.

The use of *Urtica dioica* L. has been documented as a galactogogue for cows in Italy [60]; in our survey, it was used for sprains in horses and bruises in cattle, horses, and pigs.

The widespread use of *Allium sativum* L. as vermicide has been well-documented in Romania [4], in Spain [61], in Algeria [2], and in Italy [14, 62], and our data showed its use in the treatment of intestinal worms in cattle and for hoof infection in horses and oxen. The use of garlic for bronchitis, fever, and indigestion in equines has been also reported in the Far East [63].

In the Romanian ethno-veterinary practices, *Petroselinum crispum* (Mill.) Nym has been used to improve rumination [4] while our findings showed it was used with lard and onion for bloat in cattle.

According to our interviewees, coffee would help in post-partum collapse. In Switzerland, it has been described for the treatment of gastrointestinal troubles, colic, abdominal pain, or diarrhoea [1].

The topical administration of *N. tabacum* L. leaves has been reported in our study for wounds in oxen; in Iran it was used for external and internal parasite disorders of dogs [64]; in India it was considered effective against ecto-parasites [65], while it was used for distemper, scabies, and parasitosis in Argentina [18].

In Italy, *Zea mays* L. was indicated for skin problems and wounds on cattle and for gastrointestinal complaints in horses [56], and in our survey, it was used as a remedy for indigestion in horses; in Pakistan, it was considered useful for anorexia, hematuria, weakness, and wounds in horses [63]. The use of camellia decoctions to treat wounds of horses reported in our survey cannot be found in other European or Mediterranean ethno-veterinary surveys; a traditional use of camellia in East Asia was to soothe skin [66].

Sugar has been described for the treatments of heart problem in horses in Albania [54]; Sardinian farmers, in our study, used it for cattle post-partum collapse and horse hoof infections.

Only few ethno-veterinary remedies implying the use of plants or part of plants (*Prunus spinosa*, *Allium coepa*, *Smilax aspera*, *Plantago major*, *Camellia*, *Olea europaea*, *Vitis vinifera*, *Quercus suber*), are still in use in Sardinia.

## Conclusion

The Mediterranean rural culture still maintains knowledge about many traditional herbal and non-herbal remedies for curing or treating animals, although in recent years the development of modern livestock farming technologies, administrative controls, and the denial of popular remedies have led to neglect those practices. Considering that only 27 out of the 197 reported remedies are still in use and that the knowledge was mostly hold by the most aged informants, it can be easily foreseen the loss of knowledge about such traditional ethno-veterinary practices in Sardinia. Our survey recovering ancient ethno-veterinary traditions can prevent their disappearance. It is to remark that only a few out of the 27 remedies still in use imply the utilization of plants; as a consequence, the ethno-botanic knowledge related to traditional animal care is going to be lost. The knowledge of traditional ethno-veterinary practices can be a source of useful information for the isolation of natural extracts to develop new products for health care and well-being of animals. Our data may represent novel opportunities for performing further studies, starting from ancient traditions, aimed at uncovering effective natural sources of bio-antioxidants, and new natural products for the well-being and health care of domestic animals. In agreement to Meyer-Rochow [44], the challenge is to identify those traditional healing methods that do have something to offer before nobody knows anything anymore about them and such healing methods have disappeared from the collective memory of a people.

## References

[1] Bischoff T, Vogl CR, Ivemeyer S, Klarer F, Meier B, Hamburger M, Walkenhorst M (2007). Plant and natural product based homemade remedies manufactured and used by farmers of six central Swiss cantons to treat livestock. Livest Sci.

[2] Pieroni A, Giusti M, de Pasquale C, Lenzarini C, Censorii E, Gonzáles-Tejero M, Sánchez Rojas CP, Ramiro Gutiérrez JM, Skoula M, Johnson C, Sarpaki A, Della A, Paraskeva Hadijchambi D, Hadjichambis A, Hmamouchi M, El Jorhi S, El Demerdash M, El Zayat M, Al Shahaby O, Houmani Z, Scherazed M (2006). RUBIA project: Circum-Mediterranean cultural heritage and medicinal plant uses in traditional animal healthcare: a field survey in eight selected areas within the RUBIA project. J Ethnobiol Ethnomed.

[3] Lans C, Nancy T, Gerhard B, Grant L, Karla G (2006). Ethnoveterinary medicines used for horses in Trinidad and in British Columbia, Canada. J Ethnobiol Ethnomed.

[4] Bartha SG, Quave CL, Balogh L, Papp N (2015). Ethnoveterinary practices of Covasna County, Transylvania, Romania. J Ethnobiol Ethnomed.

[5] Anyinam C (1995). Ecology and ethnomedicine: exploring links between current environmental crisis and indigenous medical practices. Soc Sci Med.

[6] Pieroni A, Howard P, Volpato G, Santoro RF (2004). Natural remedies and nutraceuticals used in ethnoveterinary practices in Inland Southern Italy. Vet Res Comm.

[7] Ali-Shtayeh MS, Jamous RM, Jamous RM (2016). Traditional Arabic Palestinian ethnoveterinary practices in animal health care: a field survey in the West Bank (Palestine). J Ethnopharmacol.

[8] Baharvand-Ahmadi B, Asadi-Samani M (2017). A mini-review on the most important effective medicinal plants to treat hypertension in ethnobotanical evidence of Iran. J Nephropharmacol.

[9] Akerreta S, Calvo MI, Cavero RY (2010). Ethnoveterinary knowledge in Navarra (Iberian Peninsula). J Ethnopharmacol.

[10] Benarba B, Belabid L, Righi K, Bekkar A, Elouissi M, Khaldi A, Hamimed A (2015). Ethnobotanical study of medicinal plants used by traditional healers in Mascara (north west of Algeria). J Ethnopharmacol.

[11] Barkaoui M, Katiri A, Boubaker H, Msanda F (2017). Ethnobotanical survey of medicinal plants used in the traditional treatment of diabetes in Chtouka Ait Baha and Tiznit (western anti-atlas), Morocco. J Ethnopharmacol.

[12] Di Sanzo P, De Martino L, Mancini E, De Feo V (2013). Medicinal and useful plants in the tradition of Rotonda, Pollino National Park, southern Italy. J Ethnobiol Ethnomed.

[13] Piluzza G, Virdis S, Serralutzu F, Bullitta S (2015). Uses of plants, animal and mineral substances in Mediterranean ethno-veterinary practices for the care of small ruminants. J Ethnopharmacol.

[14] Bullitta S, Piluzza G, Viegi L (2007). Plant resources used for traditional ethnoveterinary phytoterapy in Sardinia (Italy). Genet Resour Crop Evol.

[15] Sindhu ZUD, Ullah S, Abbas RZ, Iqbal Z, Hameed M (2012). Inventory of ethno-veterinary practices used for the control of parasitic infections in district Jhang, Pakistan. Int J Agr Biol.

[16] Yadav M, Rajput DS, Mishra P (2016). Ethno-veterinary practices among tribes of Banswara District of Rajasthan. Indian Res J Ext Educ.

[17] Kujawska M, Klepacki P, Łuczaj Ł (2017). Fischer’s plants in folk beliefs and customs: a previously unknown contribution to the ethnobotany of the polish-Lithuanian-Belarusian borderland. J Ethnobiol Ethnomed.

[18] Martínez GJ, Luján MC (2011). Medicinal plants used for traditional veterinary in the Sierras de Córdoba (Argentina): an ethnobotanical comparison with human medicinal uses. J Ethnobiol Ethnomed.

[19] Council Regulation (EC) no. 834/2007 on Organic Production and Labelling of Organic Products. http://eur-lex.europa.eu/LexUriServ/LexUriServ.do?uri=OJ:L:2007:189:0001:0023:EN:PDF. Council Regulation (EC) no. 889/2008 on laying down detailed rules for the implementation of Council Regulation (EC) no. 834/2007. http://eur-lex.europa.eu/LexUriServ/LexUriServ.do?uri=OJ:L:2008:250:0001:0084:en:PDF. Accessed 17 July 2018.

[20] Boukraa L, Benbarek H, Benhanifia M, Katerere DR, Luseba D (2010). Herbal medicines for animal health in the Middle East and North Africa (MENA) regions. Ethnoveterinary botanical medicine. Herbal medicines for animal health.

[21] Lev E (2003). Traditional healing with animals (zoo-therapy): medieval to present day Levantine practice. J Ethnopharmacol.

[22] Quave CL, Pieroni A, RRN A, Rosa IC (2013). Mediterranean zootherapy: a historical to modern perspective. Animals in traditional folk medicine: implications for conservation.

[23] Lawal OA, Banjo AD (2007). Survey for the usage of arthropods in traditional medicine in southwestern Nigeria. J Entomol.

[24] Alves RRN, Barbosa JA, Santos SL, Souto W, Barboza RR. Animal-based remedies as complementary medicines in the semi-arid region of northeastern Brazil. Evid Based Complement Alternat Med. 2011; 10.1093/ecam/nep134. 10.1093/ecam/nep134 PMC3094714 19729490

[25] Martínez GJ (2013). Use of fauna in the traditional medicine of native Toba (Qom) from the argentine Gran Chaco region: an ethno-zoological and conservationist approach. Ethnobiol Conservat.

[26] Alonso-Castro AJ (2014). Use of medicinal fauna in Mexican traditional medicine. J Ethnopharmacol.

[27] Dossey AT (2010). Insects and their chemical weaponry: new potential for drug discovery. Nat Prod Rep.

[28] Alves RRN, Rosa IL (2005). Why study the use of animal products in traditional medicines?. J Ethnobiol Ethnomed.

[29] González JA, Amich F, Postigo-Mota S, Vallejo JR (2016). Therapeutic and prophylactic uses of invertebrates in contemporary Spanish ethnoveterinary medicine. J Ethnobiol Ethnomed.

[30] Menale B, De Castro O, Cascone C, Muoio R (2016). Ethnobotanical investigation on medicinal plants in the Vesuvio National Park (Campania, southern Italy). J Ethnopharmacol.

[31] Wilkens B (2004). La fauna sarda durante l’olocene: le conoscenze attuali. Int J Archeol.

[32] Mascheroni E (1929). Zootecnia speciale. Equini.

[33] Gratani L (1984). Cavalli di Sardegna. Regione Autonoma della Sardegna, Assessorato Agricoltura e Riforma Agro-pastorale, Istituto Incremento Ippico della Sardegna.

[34] Viegi L, Bioli A, Vangelisti R, Cela Renzoni G (1999). Prima indagine sulle piante utilizzate in medicina veterinaria popolare in alcune localita` dell’alta Val di Cecina. Atti Soc Tosc Sci Nat Mem Ser B.

[35] Pignatti S (1982). Flora d’Italia.

[36] Conti F, Abbate G, Alessandrini A, Blasi C (2005). An annotated checklist of the Italian vascular flora.

[37] Angiosperm Phylogeny Group (2016). An update of the angiosperm phylogeny group classification for the orders and families of flowering plants: APG IV. Bot J Linn Soc.

[38] Tardío J, Pardo-de-Santayana M (2008). Cultural importance indices: a comparative analysis based on the useful wild plants of Southern Cantabria (Northern Spain). Econ Bot.

[39] Pardo-de-Santayana M. Las plantas en la cultura tradicional de la antigua Merindad de Campoo. Ph.D. dissertation, Departamento de Biología, Facultad de Ciencias, Universidad Autónoma de Madrid, Spain. 2003.

[40] Fois P, Mura L, Bullitta S (2000). Plant genetic resources protection in the Mediterranean basin: the case of Sardinian forage species. Cah Options Méditerranéennes.

[41] Bullitta S, Swiecicki W, Naganowska B, Wolko B (2001). Legal protection of local genetic resources and regulations for germplasm collection activities for scientific, economic and commercial purposes. Broad variation and precise characterization-limitation for the future. Eucarpia section genetic resources.

[42] Voultsiadou E (2010). Therapeutic properties and uses of marine invertebrates in the ancient Greek world and early Byzantium. J Ethnopharmacol.

[43] Ahmad TB, Liu L, Kotiw M, Benkendorff K. Review of anti-inflammatory, immune-modulatory and wound healing properties of molluscs. J Ethnopharmacol. 2017; 10.1016/j.jep.2017.08.008. 10.1016/j.jep.2017.08.008 28830818

[44] Meyer-Rochow VB (2017). Therapeutic arthropods and other, largely terrestrial, folk-medicinally important invertebrates: a comparative survey and review. J Ethnobiol Ethnomed.

[45] Quave CL, Pieroni A, Bennet CB (2008). Dermatological remedies in the traditional pharmacopoeia of vulture-alto Bradano, inland southern Italy. J Ethnobiol Ethnomed.

[46] Confessor MV, Mendonça LE, Mourão JS, Alves RR (2009). Animals to heal animals: ethnoveterinary practices in semiarid region, northeastern Brazil. J Ethnobiol Ethnomed.

[47] Alves RRN, Alves HN (2011). The faunal drugstore: animal-based remedies used in traditional medicines in Latin America. J Ethnobiol Ethnomed.

[48] Volpato G, Kourková P, Zelený V (2012). Healing war wounds and perfuming exile: the use of vegetal, animal, and mineral products for perfumes, cosmetics, and skin healing among Sahrawi refugees of Western Sahara. J Ethnobiol Ethnomed.

[49] Jarald E, Edwin S, Tiwari V, Garg R, Toppo E (2008). Antioxidant and antimicrobial activities of cow urine. Glob J Pharmacol.

[50] Tulobaev AZ, Aldaiarov N, Jumakanova Z, Niiazbekova Z. Information on traditional veterinary knowledge of Kyrgyz people. Manas journal of agriculture and veterinary life. Science. 2016;6(2):29–35

[51] Yadav ML, Rajput DS (2015). Ethno-veterinary practices by tribals of Banswara district of Rajasthan. Indian J Nat Prod Res.

[52] Maraver F, Fernández-Torán MÁ, Corvillo I, Morer C, Váquez I, Aguilera L, Armijo F (2015). Pelotherapy, a review. Med Nat.

[53] Volpato G, Saleh SML, Nardo A (2015). Ethnoveterinary of Sahrawi pastoralists of Western Sahara: camel diseases and remedies. J Ethnobiol Ethnomed.

[54] Pieroni A, Nedelcheva A, Hajdari A, Mustafa B, Scaltriti B, Cianfaglione K, Quave CL (2014). Local knowledge on plants and domestic remedies in the mountain villages of Peshkopia (eastern Albania). J Mt Sci.

[55] Ghazanfar SA (1995). Wasm: a traditional method of healing by cauterization. J Ethnopharmacol.

[56] Viegi L, Pieroni P, Guarrera PM, Vangelisti R (2003). A review of plants used in folk veterinary medicine in Italy as basis for a databank. J Etnopharmacol.

[57] Atzei AD (2003). Le piante nella tradizione popolare della Sardegna.

[58] González JA, García-Barriuso M, Amich F (2011). Ethnoveterinary medicine in the Arribes del Duero, western Spain. Vet Res Commun.

[59] Bonet MA, Vallès J (2007). Ethnobotany of Montseny biosphere reserve (Catalonia, Iberian Peninsula): plants used in veterinary medicine. J Ethnopharmacol.

[60] Cornara L, La Rocca A, Terrizzano L, Dente F, Mariotti MG (2014). Ethnobotanical and phytomedical knowledge in the north-western Ligurian alps. J Ethnopharmacol.

[61] Blanco E, Macía MJ, Morales R (1999). Medicinal and veterinary plants of El Caurel (Galicia, Northwest Spain). J Ethnopharmacol.

[62] Guarrera PM, Lucchese F, Medori S (2008). Ethnophytotherapeutical research in the high Molise region (central-southern Italy). J Ethnobiol Ethnomed.

[63] Goraya K, Iqbal Z, Sajid MS, Muhammad G, ul Ain Q, Saleem M (2013). Diversity of flora used for the cure of equine diseases in selected peri-urban areas of Punjab, Pakistan. J Ethnobiol Ethnomed.

[64] Bahmani M, Eftekhari Z (2013). An ethnoveterinary study of medicinal plants in treatment of diseases and syndromes of herd dog in southern regions of Ilam province, Iran. Comp Clin Pathol.

[65] Adhikary SP (2014). Indigenous knowledge on animal care practices in Surada block of Ganjam District, Odisha. Eur J Environ Health Ecol.

[66] Lim TK (2014). Camellia japonica. Edible medicinal and non-medicinal plants.

